# A Case-Control Study of Paracoccidioidomycosis in Women: The Hormonal Protection Revisited

**DOI:** 10.3390/jof7080655

**Published:** 2021-08-13

**Authors:** Tereza Graciano Nascimento de Brito, Mariane Taborda, Bruna Provenci, André Nathan Costa, Gil Benard

**Affiliations:** 1 Laboratorio de Investigacao em Imunologia e Dermatologia (LIM56), Departamento de Dermatologia and Instituto de Medicina Tropical, Faculdade de Medicina, Universidade de Sao Paulo, SP, R. Dr. Enéas de Carvalho Aguiar 470, São Paulo 05403-000, Brazil; tereza.brito@uscsonline.com.br; 2 Divisao de Doenças Infecciosas, Hospital das Clinicas, Faculdade de Medicina, Universidade de Sao Paulo, SP, R. Dr. Enéas de Carvalho Aguiar 255, São Paulo 05403-000, Brazil; mary861@gmail.com; 3 Divisao de Pneumologia, Instituto do Coração, Hospital das Clinicas, Faculdade de Medicina, Universidade Sao Paulo, SP, R. Dr. Enéas de Carvalho Aguiar 44, São Paulo 05403-900, Brazil; bruna_provenci@hotmail.com (B.P.); nathan.andre@gmail.com (A.N.C.); 4 Laboratorio de Micologia Medica (LIM53), Departamento de Dermatologia and Instituto de Medicina Tropical, Faculdade de Medicina, Universidade de Sao Paulo, SP, R. Dr. Enéas de Carvalho Aguiar 470, São Paulo 05403-000, Brazil

**Keywords:** paracoccidioidomycosis, estrogens, female, susceptibility, skin test

## Abstract

Clinical observations have long suggested that women are protected against paracoccidioidomycosis. 17β-estradiol, the main female estrogen, inhibits conidia-to-yeast transformation (C-to-Y), which is required for the infection establishment. However, experiments in murine models have yielded conflicting results, suggesting that C-to-Y inhibition, alone, fails to explain the female-associated protection and that sexual hormones may also act by modulating the host’s immune responses. Therefore, this issue remains unsolved. Strikingly, no studies have compared the severity of paracoccidioidomycosis between men and women. This retrospective case-control study compared 36 women with 72 age-matched men for clinical–demographic, laboratory, and chest imaging findings. Overall, paracoccidioidomycosis in women presented the main features described in the acute/subacute and chronic forms seen in men. Women also showed similar demographic features and clinical–laboratory and imaging severity scores as men. We additionally reviewed 58 paracoccidioidin skin test surveys undertaken by volunteers from endemic areas. Data accumulated from 10.873 tests showed that females and males are infected with similar magnitudes (21.9% vs. 25.2%) and that reactivity steadily increased with age, peaking after the age of 60. We discuss the paradox of similar infection rates but much lower disease prevalence in women, considering the current pathogenetic views of paracoccidioidomycosis, and we raise alternative hypotheses to account for this paradox.

## 1. Introduction

Paracoccidioidomycosis (PCM) is the most important systemic mycosis in Brazil and also an important public health issue in other parts of Latin America. It is caused by the dimorphic fungi of the genera *Paracoccidioides*, estimated to dwell in the soil of the endemic areas in its filamentous form [1]. Patients usually report living or having lived in rural areas and to have exerted land-related activities. The disease is acquired through inhalation of conidia produced by the filamentous form of the fungi. Once they reach terminal bronchi or alveolar spaces, they transform into yeast cells, which then can spread through the lymphohematogenous route. The establishment of the infection depends on pathogenic features of the fungus as well as the host’s anti-*Paracoccidioides* immune status, particularly in relation to the cell-mediated immune responses [1]. The latter should result in the formation of granulomas, which at least partially contain fungus spread. The balance in host–parasite interaction will dictate the outcome of the infection, from asymptomatic or subclinical infection to the development of disease with few or many foci and with variable severity. Factors estimated to affect disease severity or susceptibility are nutritional deficits, immunosuppression (e.g., HIV infection, corticosteroids, chemotherapy, transplantation), tobacco exposure, and alcohol abuse. Two main clinical presentations are considered: the acute/subacute form (A/SAF), rare, and the chronic form (CF), developed by ≥90% of the patients. The A/SAF affects mainly children to young adults under the age of 30, usually weeks to several months after the initial infection, and is characterized by the widespread involvement of the lymphatic system. The CF, on the other hand, arises from subclinical foci established many years earlier during the initial infection, causing predominant involvement of the respiratory and oropharyngeal mucosa in adult males [2].

Surveys with *Paracoccidioides* spp. skin tests suggest that there are no gender differences in the rate of positive tests in endemic areas [3], leading to the assumption that the acquisition of *Paracoccidioides* spp. infection occurs equally in men and women (and also in boys and girls). This is in sharp contrast with the gender difference in disease prevalence. Before puberty, PCM (disease) tends to affect both sexes more closely. This is well illustrated in the study by Romaneli et al., which showed that, in children up to 12 y/o, the male-to-female ratio was 1.5:1, while male predominance started in children between 12 and 15 y/o (7:1) [4]. In studies of large cohorts consisting predominantly of adults, women corresponded to less than 5% of the cases [5,6,7,8]. The basis for the resistance of adult women in developing PCM was studied mainly by the groups of Restrepo A. and Stevens D. They showed in a number of elegant in vitro and mice PCM models (reviewed in [3]), that 17β -estradiol at physiological levels binds to a specific cytosolic receptor of *Paracoccidioides* conidia and hyphae forms, thereby inhibiting their conversion to the parasitic yeast form, a key step in the pathogenesis of the mycosis. It was also shown that this binding induced a re-programming of gene expression of the fungus, in particular of signaling genes that regulate dimorphism, thus acting as a true receptor. Interestingly, however, experiments in which mice models of PCM were castrated and then hormonally reconstituted with their own or opposite sexual hormone, supported, on one hand, the in vitro findings of conidia to yeast (C-to-Y) transformation inhibition and subsequent resistance of females to develop severe disease. On the other hand, it showed that possibly 17β-estradiol, alone, is not sufficient to prevent infection establishment [9]. The authors suggested that, eventually, sexual hormones could also influence the host’s immune response [3].

Therefore, this issue still remains unsolved. Strikingly, however, there are no studies comparing the severity of the disease between adult men and women with PCM.

## 2. Patients and Methods

The study was approved by the Hospital’s Ethics Committee (No. 3.079.825/2018). This was a retrospective case-control study of two cohorts of PCM patients admitted at the Instituto Central do HCFMUSP between 2001 and 2018 (from 2001 onward, laboratory and imaging records of the patients began to be stored online). First, women with a diagnosis of PCM within this period were identified in the hospital files/archives. Thirty-five cases were retrieved. Eleven were classified as having the A/SAF and twenty-four as having the CF. For each woman, we then searched for two corresponding men with the same clinical form of PCM diagnosed at the same time period and matched for age (±2 years) (*n* = 70, 22 with A/SAF and 48 with CF). This was done in a blind fashion using the hospital’s registry code (six numbers and one letter) by one of the authors (T.G.N.d.B.) who does not have clinical activity at the hospital. In all cases, the diagnosis of PCM was done based on clinical and epidemiological grounds plus identification of typical forms in appropriately stained patients’ specimens or positive serological tests (a positive immunodiffusion test and a titer >1:8 in the counterimmunoelectrophoresis test) [10]. 

Demographic and comorbidity data were collected from all cases. To compare the severity of the disease between women and men, we used clinical, laboratory, and imaging (pulmonary HRCT scans in CF patients) data. The selected clinical parameters were fever, weight loss, jaundice, splenomegaly, hepatomegaly, oral or nasal mucosal lesions, laryngeal involvement, lymph node enlargements (cervical, supraclavicular, axillary, thoracic, abdominal, and inguinal), skin lesions, lung involvement, osteoarticular involvement, and central nervous system (CNS) involvement. A clinical score was calculated, with 0 for absence and 1 for the presence of abnormalities, from fever to laryngeal involvement; the presence of the last four types of involvement (from skin lesions to CNS involvement) was arbitrarily scored 2 due to the fact that they represent more severe or extensive involvement or hematogenic spread. For the lymph node enlargements, each localization was scored 1. The maximum possible clinical score was 21. The adrenal reserve was not included since it was not systematically investigated. The following laboratory parameters were selected, all of which were usually considered as correlates of disease severity [11]: counterimmunoelectrophoresis serology titers, albumin and gamma globulin levels, albumin/gamma globulin ratio, hemossedimentation rate, reactive-C protein, leukocytosis, and leukopenia. Liver function tests were not included in the comparison, because significantly more male patients reported alcohol abuse. Gynecologic data and hormone dosage were not available in most women and were not considered. They were classified as within or outside of childbearing age according to WHO [12].

A thoracic high-resolution computed tomography (HRCT) done prior to or at the start of the treatment was available in 34 patients, 21 men and 13 women. Many exams were done only a few months after the initiation of the treatment, especially in nonhospitalized patients. The images were analyzed by two investigators blind to the patients’ clinical history, considering the types of radiological findings. Table 1 lists the 16 types of findings analyzed.

Statistical analyses were carried out using Prisma software (GraphPad Software, Inc., San Diego, CA, USA). The Mann–Whitney and ANOVA with Dunn’s post-test and Fisher exact test were used. The study was approved by the Hospital’s Institutional Board (No. 3.079.825/2018).

## 3. Results

### 3.1. Clinical and Demographic Characteristics of Women with PCM

**Figure 1:**
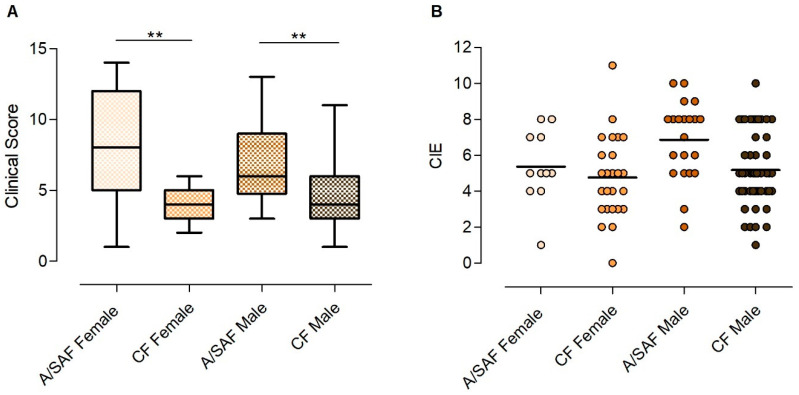
(**A**) Clinical score of the paracoccidioidomycosis patients on admission, according to the clinical form and gender (acute/subacute form (A/SAF) and chronic form (CF)). Medians, interquartile ranges, and min./max. ** *p* < 0.01. (**B**) Quantitation of anti-*Paracoccidioides brasiliensis* antibody titers by counterimmunoelectrophoresis (CIE) in the serum of paracoccidioidomycosis patients on admission, according to the clinical form and gender. Each dot represents a patient; the lines denote medians.

Overall, PCM in women presented the main features described in large cohorts comprised mostly of men, with a clear distinction between the A/SAF and the CF. In both forms, there was a history of present or past link with rural areas, an association with tuberculosis in up to 10% of the cases, and a presence of other serious comorbidities that may have eventually caused a predisposition to mycosis, particularly cancer (Table 1). Strikingly, there was an almost absolute requirement of a cigarette smoking habit in the CF, while alcohol abuse, relatively common in PCM (~60%) [13], was infrequent. Moreover, consistent with our current knowledge of the mycosis, clinical manifestations of the A/SAF were more severe or disseminated, as indicated by the more frequent presence of for example, fever, emaciation, icterus, skin, and lymph node involvement, while the CF was characterized by upper and lower respiratory tract mucosa involvement (Table 2). This resulted in a significantly higher clinical score in the A/SAF than in the CF (Figure 1A).

In order to determine whether sexual hormones play a role in the severity of the clinical manifestations and laboratory parameters, we compared age-matched men and women with PCM who were admitted at our institution between 2001 and 2017. Patients with the A/SAF and the CF were compared separately.

### 3.2. Comparison between the A/SAF of PCM in Males and Females

No differences between A/SAF male and female patients were found regarding demographic data (Table 1). Of the 13 distinct clinical manifestations searched, there was an overlap between both sexes in 12 of those, with the single exception that women registered weight loss more frequently (Table 2). Some other findings such as hepatomegaly, splenomegaly, and jaundice were somewhat more frequent in women than men, but they did not reach statistical significance. However, the average clinical score based on these manifestations showed no significant difference between the two sexes (Figure 1A). Of the nine laboratory parameters investigated that correlate with disease severity (e.g., hypergammaglobulinemia, hypoalbuminemia, C-reactive protein, etc.), all were comparably altered in both sexes except for anemia, which was more frequently detected in women (Table 3). Finally, serology titers were strongly positive and not significantly different between both sexes (Figure 1B). Overall, both men and women presented comparable signs and symptoms of a relatively severe systemic disease, accompanied by elevated levels of inflammatory markers and anti-*Paracoccidioides* antibody titers.

### 3.3. Comparison between the CF of PCM in Males and Females

Although the overall demographic background was similar between both sexes, as for the A/SAF, an important difference was noted. While a diagnosis of prior tuberculosis, cancer, or other comorbidities was occasionally found in both groups/sides, and the presence of a smoking habit and rural exposure were the rule, the proportion of men in reference to alcohol abuse was much higher than in women (*p* < 0.0055). There were, in fact, some differences in the clinical expression, such as a higher frequency of fever and cutaneous lesions in men. Despite these differences, the average clinical score was similar in the two groups. Regarding laboratory work, males displayed anemia, hypoalbuminemia, and elevated CRP more frequently than women. Other parameters were also frequently abnormal but in a similar fashion in the two groups. Anti-*Paracoccidioides* antibody titers were equally elevated in both groups. Thus, in both the A/SAF and CF patients examined here, we could not detect a sizable gender-related difference in the severity of the mycosis.

**Figure 2:**
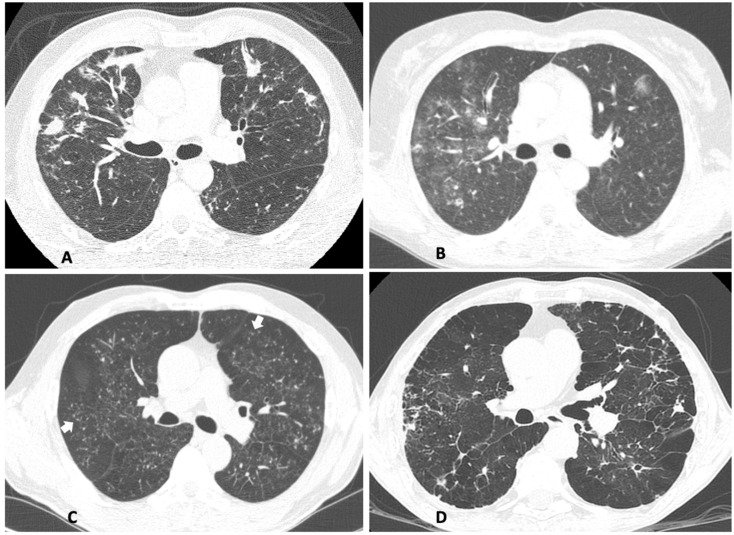
The most common alterations found on women’s pulmonary HRCT taken before treatment. (**A**) Upper lobes showing nodules. (**B**) Upper lobes showing ground-glass opacities. (**C**) Lower lobes showing micronodules (some indicated by arrows). (**D**) Diffuse emphysema and architectural distortion.

As the lungs are the main target in the CF of PCM, present in at least 80% of the cases (as was the case with our patients), a detailed scoring of the parenchymal and pleural lesions in the HRCT scan was used to infer the severity of this involvement. CT scans were available for 13 women and 21 men. At least one abnormal pulmonary finding was observed in all 34 patients for which scans were available. Nodules, ground-glass opacities, and micronodules, typically the result of the injury by the infectious process, were the most common findings, present in ~two-thirds of the patients, while the other most common finding was emphysema, probably linked mainly to cigarette smoking. Interestingly, women presented more frequently (*p* < 0.05) ground-glass opacities while men displayed more frequently (*p* < 0.05) emphysema, the latter likely due to the fact that men were exposed to a higher tobacco load than women [14]. Pleural involvement was uncommon. Other findings are described in Table 4. On average, there were 5.8 ± 2.6 abnormalities in men’s and 5.2 ± 2.1 in women’s HRCTs. Figure 2 illustrates the four most common findings in women’s HRTCs. 

No follow-up analyses were carried out, because there was better compliance to treatment and follow-up by female patients. 

### 3.4. Comparison between the CF of PCM in Females at and Not at Childbearing Age

Hormonal influence on PCM severity was further assessed by comparing the female patients at and not at childbearing age. This was done only with the CF group since almost all A/SAF patients were of childbearing age. Ten CF women were 33–45 y/o (40 ± 4.3) and 15 were 49–68 y/o (56.4 ± 5.7). Interestingly, no significant differences between the two subgroups of CF patients were found regarding the clinical score (3.6 ± 1.4 vs. 4.0 ± 1.3, respectively, *p* = 0.47), serology titers (5.3 ± 1.7 vs. 4.4 ± 2.6, *p* = 0.35) or lung HRCT score (4.5 ± 1.0 vs. 5.4 ± 2.4, *p* = 0.47).

### 3.5. Review of Skin Test Surveys with Paracoccidioides spp. Antigens in the General Population

The current view states that opposite to the high male:female PCM-disease ratio, there is no gender difference in the rate of reactors to skin tests with *Paracoccidioides* spp. preparations among volunteers in endemic areas [3]. However, there is not a thorough review of the numerous skin test surveys that have been performed since these tests were standardized in the late 1950s [15]. Fava Netto and Fava Netto reviewed this subject in 1998, retrieving 38 paracoccidioidin surveys carried out in Brazil, but did not assess gender differences [16]. We revisited these 38 studies and included 19 additional ones that were either carried out outside of Brazil or published thereafter (a total of 57) (unpublished data). Unfortunately, we could not assess eight of those studies. Of the 49 studies, 10 were excluded for this purpose, because they addressed specific populations, only (children or adolescents, soldiers, gold miners, asylum and orphanage residents, etc.) and 8 for not providing gender information.

Of the remaining 31 surveys, 18 presented no significant differences between genders [17,18,19,20,21,22,23,24,25,26,27,28,29,30,31,32,33,34], 3 displayed higher rates of positive tests in women [35,36,37], and 10 identified higher rates in men [38,39,40,41,42,43,44,45,46,47]. However, of the latter ten surveys, three mentioned a difference, but none of them provided statistical analysis nor detailed data to allow statistical manipulation; three actually showed no statistically significant difference; and, notably, of the remaining four surveys that showed a statistical difference, in one of them the percentage of positive tests in men was around only twice that of women (data not shown). On the other hand, of the three surveys referring to higher rates in women, one showed that the difference was statistically significant, the other mentioned the difference but did not provide the data or statistical analysis, and the last actually did not show a statistically significant difference. Summing up all studies that provided sufficient data, a total of 5.239 females were surveyed, of which 1.149 skins tested positive (21.9%), while, of the 5.634 men that were surveyed, 1.495 skins tested positive (26.5%). Thus, although slightly higher in men, the rates of skin test positivity in men and women are of the same magnitude. 

**Figure 3:**
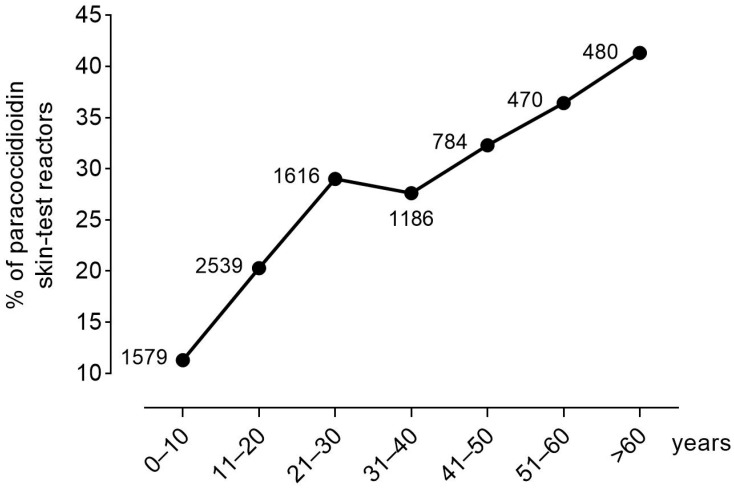
Percentage of paracoccidioidin skin test reactors gathered from references 18, 19, 21–25, 27, 28, 30, 32, 35, 36, 39–41, 44, and 47. The total number of persons tested for each age interval is indicated.

Another currently stated notion is that infection in endemic areas occurs predominantly or frequently early in life (e.g., “first two decades of their life”) [11]. However, this concept is not consistent with the results of the surveys encompassing a wide age range of the general population: the proportion of skin test reactors steadily increased with age (Figure 3). For example, in a survey carried out using gp43 (the *P. brasiliensis*-complex immunodominant and specific component) in 695 persons from a highly endemic county, the percentage of skin test reactors at early ages (2–10 y/o) was 11.1% but reached 62.1% among individuals older than 60 y/o [30]. This clearly signals that the acquisition of infection starts in childhood but increases sharply with age, denoting that those residing in endemic areas are continuously being (re)infected throughout life, especially during adulthood. 

## 4. Discussion

Clinical observations have long suggested that female sex hormones could play a protective role in the development of PCM. Reports on large cohorts of PCM patients, comprised mainly of CF cases (≥90%), the most common clinical presentation of the disease, showed a high male-to-female ratio, ranging from 5:1 to up to 22:1 [5,6,7,8]. However, they also showed that children characteristically presented the A/SAF and, in this case, the ratio approached 1:1. It was then assumed that exposure and infection with the fungus would occur at young ages, likely before puberty in most cases, when it can (rarely) cause the A/SAF in susceptible persons of both genders, while, most commonly, the disease’s CF arises many years later, when the patient attains reproductive age and estrogens protect the women. Investigators described a potential mechanism behind the predominance of the disease’s CF in males over the age of 20. They showed that 17β-estradiol, the main female estrogen, binds to a cytoplasmatic receptor of *Paracoccidioides* conidia and mycelia and inhibits their transformation into the parasitic form required for the establishment of the infection [3,9,48]. 

First, we show here that the distinct features of the A/SAF and CF, established from large cohort studies comprising mostly males, were equally observed in the 36 adult women evaluated here, 11 with the A/SAF and 25 with the CF. The clinical and demographic features commonly ascribed to the A/SAF and CF of PCM were also present in women, except for alcohol abuse, more common in men, likely due to sociocultural factors/determinants. Notably, the quasimandatory association between the CF and smoking was also present in women. As expected, clinical scoring also showed the A/SAF to be more severe than the CF in women. Second, contrary to our initial expectation of less severe disease in women, the severity of the disease was largely comparable between males and females matched for age and clinical form, using either clinical, laboratory, or imaging parameters. 

To our knowledge, a single previous communication addressed PCM in women, reporting on 23 CF patients [49]. These women were either at childbearing age, perimenopause, or menopause. Of the former, some had comorbidities that could have predisposed them to the development of the disease (AIDS and corticosteroids at an immunosuppressive dose), but most did not or, alternatively, had entered early menopause. However, a detailed gynecologic investigation, as in this study, was not available. Although they suggest that the disease in women is related to the peri- or postmenopausal period due to estrogen protection, no mechanisms were provided for the development of the disease in said women at childbearing age.

On the contrary, some important issues are not considered when using the C-to-Y conversion mechanism to explain women’s protection against PCM. First, the CF arises from subclinical foci containing small proportions of replicating yeast cells amidst predominantly quiescent cells, a process that can take several years or even decades [50]. Thus, even in women who developed the CF of PCM at the perimenopause, the infection was acquired much earlier, i.e., before puberty, because thereafter estrogens would have halted the infectious process. However, this reasoning does not hold true for two major reasons. First, because paracoccidioidin skin test surveys not only show that women are infected at almost the same rate as men in endemic areas but also that the infection or reinfection(s) happens throughout life, increasing steadily with age to reach over 40% of the tested subjects by the age of 60, while before puberty the proportion is only ~10% (see Figure 3). Thus, the arising question is why estrogen protection fails to abrogate the steady increase in the rate of infection with age, particularly during women’s childbearing period of life. Second, opposite to conidia and mycelia, it was demonstrated that estrogens do not affect *Paracoccidioides* yeast cells, affecting neither their metabolism, growth, nor replication [3]. Once women are infected and remain with subclinical foci-containing yeast cells, these cells would be, as in men, released to replicate and cause disease at any age. Thus, why should menopause be a risk factor for the CF disease since estrogens do not affect yeast cells? In conclusion, the mechanism of hormonal protection, alone, as currently proposed (blockade of C-to-Y transformation) fails to explain the disease’s features in women, as has been previously identified [3].

While our cohort, on one hand, confirms the rarity of PCM in women, it also shows that the CF occurs in women either before or at/after the perimenopause: they were all older than 32 y/o, and 40% were at childbearing age. Interestingly, a comparison of the clinical, laboratory, and lung HRCT scores between the CF of PCM women at childbearing age and perimenopause showed no differences, challenging the role played by estrogens in curtailing the severity of the disease in women. Moreover, if this were true, women with the CF of PCM would be older than men. However, the mean age of our group of CF women (49.8 ± 9.8 y/o) was not older but rather similar to that reported for large cohorts of CF male patients, which had an average of 47–49 y/o at diagnosis [5,6,7,8]. In the study of Severo et al. [49], the mean age (51.0 ± 9.6, *p* = 0.6) was also not older than that of our CF women.

The influence of gender on the A/SAF was also analyzed. The number of patients was small, but the results pointed in the same direction as in the CF: no major differences in comorbidities and epidemiological features, the severity of clinical manifestations, or pattern of laboratory markers of severity could be found between age-matched A/SAF male and female patients. Strikingly, our A/SAF women were at least 18 y/o and 82% were at childbearing age. Based on the facts that (i) the incubation period of the A/SAF is much shorter than that of the CF, weeks to months (although a few years cannot be ruled out in some cases) and (ii) the menarche in Brazil is around 12 y/o [51], all patients have been infected when estrogens were present. These, therefore, should have halted the C-to-Y conversion and abrogated the establishment of the infection. However, this did not happen.

What would then be the role of estrogens in the natural history of PCM? Experimental studies have only partially helped to clarify this issue. Mice and other animal models in which yeast cells were inoculated showed mixed results, either no difference between males and females or females were more resistant [3], as well as a study using high-inoculum doses of conidia via an intrapulmonary route that showed more severe pathology in females than males [52]. In this case, it was later argued that the anesthetic used (diethyl–ether) could have influenced the results, as it damages the alveolar lining. Studies with castrated mice inversely hormonally reconstituted also revealed inconsistent results. Male castrated mice reconstituted with 17β-estradiol and infected through the intranasal route with conidia initially controlled the infection but subsequently developed high fungal burdens, even higher than those of castrated male mice not reconstituted with the estrogen or those of castrated females [9]. 

An alternative possibility is that the hormonal protection would be overcome by a higher frequency of comorbidities impacting the immune system, leading to an imbalance of the anti-*Paracoccidioides* immune response and clearing the way to disease onset. This was not verified in our study since there were no marked differences in the type and number of associated comorbidities between males and females. Important limitations of our study, however, are the lack of appropriate gynecological investigation of the patients and the relatively small number of women with PCM assessed, especially the A/SAF, the latter maybe underpowering the statistical analyses.

On the other hand, sexual hormones can also regulate immune responses, biasing the immune reactivity of males and females [53,54]. Based mainly on experimental data, it has been suggested that feminine hormones would favor cell-mediated immunity by enhancing Th-1 immune responses and macrophage function, while testosterone would do the reverse. A study showed differences in innate and acquired immune responses of female and male mice infected intraperitoneally with yeast cells. Although the model did not mimic the human infection and the study focused mainly on the responses to a *Paracoccidioides* lectin, the fungal burden was reduced in females, which also displayed better Th-1 responses and macrophage killing activity [55].

Based on the rationale that women have better cell-mediated immunity [53,54], one would expect those chronic infectious diseases that require a Th-1/granulomatous response to their control to be either more frequent (or more severe) in males than females. This indeed holds true with the systemic mycoses germane to PCM, histoplasmosis, coccidioidomycosis, and blastomycosis, as well as tuberculosis. In tuberculosis, the best-studied granulomatous infectious disease has an incidence of around twice in men than women [56]. This bias, nevertheless, is generally ascribed to the higher risk of men developing the disease due to sociocultural determinants, such as behaviors leading to higher exposure, higher rates of cigarette smoking and alcohol abuse, etc. [57,58]: gender-associated immune response bias is not usually considered [59]. This reasoning agrees with the higher rates of positive tuberculin responses in men than women [60]. Similarly, blastomycosis, coccidioidomycosis, and histoplasmosis are all more common in men: in none of them, however, the M:F bias reaches the magnitude of PCM, being estimated as 1.5–2:1, 3–4:1, and 2:1, respectively [61,62,63]. The grounds for this small but sustained consistent M:F bias in these mycoses is poorly discussed, being occasionally ascribed to gender-associated differences in environmental exposure.

Our hypothesis to explain the high M:F ratio in the CF of PCM considers several factors. Part of the women’s protection certainly relies on the inhibition of the C-to-Y transformation in the lungs. As the skin test surveys suggest, persons living in endemic areas are frequently being infected/reinfected, probably with small inocula. This is consistent with the findings of studies showing frequent molecular detection of *Paracoccidioides* spp. in environmental air and soil samples collected from endemic sites as well as with serological surveys of different mammalian species (e.g., sheep, horses, dogs) of rural areas in Brazil showing high rates of anti-*Paracoccidioides* antibody responses and low rates in animals from urban areas [64,65,66,67,68,69]. Overall, these observations suggest that exposure to *Paracoccidioides* spp. does not seem to be a rare event. It is estimated that only a small fraction of the exposed men is successfully infected, i.e., the inhaled conidia convert into yeast cells in the lower respiratory tract, followed by an invasion of the epithelial lining, with the formation of subclinical foci containing live yeast cells, a proportion of them retaining replicative activity, as previously outlined [48]. In the rare instance/occasion that the host does not mount appropriate anti-*Paracoccidioides* specific immune responses, the A/SAF develops [70]. Otherwise, these foci are at least partially controlled by the cell-mediated immune response, with the reinfection episodes helping to sustain this immunity, as witnessed by paracoccidioidin skin test reactivity, avoiding the development of overt disease and eventually sterilizing the foci. This course of events can nevertheless be modulated by factors that disturb the host–parasite balance, such as cigarette smoking, alcohol abuse, undernutrition, and others, allowing the gradual progression of the mycologically active subclinical foci to the CF of the disease. 

How, then, would the course of events aforementioned apply to women at childbearing age (and at perimenopause)? According to experiments in female mice, inhaled conidia can survive in the airspaces for up to 96 h without yeast transformation [9]. We hypothesize that these conidia would be able to cross the epithelial barrier, elicit an immune response mediated mainly by macrophages (and eventually DC), while PMN would be the main cell type in males [50], priming more efficient T-cell responses. The continuous re-exposure to conidia also sustains a robust cell-mediated immunity, translated into persistently positive paracoccidioidin skin tests. In these cases, the (re)infection results in self-limited inflammatory foci without active yeast cells. This hypothesis implies that the women who develop the CF of PCM at childbearing age must have been infected before puberty, remaining for a very long period (decades) with subclinical but mycologically active foci. This would be an unusual event but as such in agreement with the fact that the CF disease is unusual in women postpuberty. Notably, almost all women with the CF in our study were smokers, a condition that strongly predisposes them to the CF [13]. Women in general (i) less frequently smoke (in Brazil, the M:F ratio being around 2:1) and (ii) smoke less (lower pack/years rates) [14], both contributing further to the rarity of the CF, even though ~22% of the women in endemic areas are infected with *Paracoccidioides* spp. Finally, an additional aspect contributing to the male bias is that the host–parasite balance would be more susceptible to immune modulation in men than women, based on the findings that estrogens favor better cell-mediated immune responses, such as enhanced Th-1 cytokines secretion by T-cells and macrophage phagocytosis and nitric oxide release [53,54].

The mechanisms that underlie the development of the A/SAF in childbearing aged women who have likely been infected a short time before the emergence of clinical manifestations, thus under the influence of estrogens, remain to be determined. The mechanisms may be related to those that underlie the paradoxical high fungal burdens that appeared in castrated male mice reconstituted with 17β-estradiol [9]. Further studies with larger cohorts of women with paracoccidioidomycosis are warranted, not only to elucidate the paradox between rates of infection as high as those in men with much lesser susceptibility to the disease but also to provide new insights on the still poorly known pathogenesis of the mycosis.

## Figures and Tables

**Table 1:** Epidemiological data of women and men with paracoccidioidomycosis.

	FEMALE		MALE	
Epidemiologic Data	Acute/Subacute Form	Chronic Form	Acute/Subacute Form	Chronic Form
Age	29.8 ± 3.7	49.8 ± 2	29.4 ± 1.8	51.8 ± 1.2
Exposure to Rural Area	8/9 88.9%	19/20 95%	14/1 93.3%	39/42 92.9%
Cigarette Smoking	4/11 36.4%	23/24 95.8%	11/22 50%	50/50 100%
Alcohol Abuse	0/11 0%	4/25 ** 16%	3/22 13.6%	25/50 ** 50%
Tuberculosis	1/11 9%	2/25 8%	1/22 4.5%	5/50 10%
Cancer	1/11 9%	4/25 16%	0/22 0%	4/50 8%
Other Comorbidities ^a^	1/11 9%	8/25 32%	0/22 0%	11/50 22%
Childbearing Age	9/11 * 81.8%	10/25 * 40%	NA	NA

* *p* < 0.05, ** *p* <0.01. ^a^ Chronic Obstructive Pulmonary Disease (*n* = 11), Diabetes Mellitus (5), HIV (1), Hypothyroidism (1), Leprosy (1), Systemic erythematous lupus (1). NA, not applicable. Some information was missing in certain patients.

**Table 2:** Main clinical manifestations of women and men with paracoccidioidomycosis.

	FEMALE		MALE	
Clinical Manifestation	Acute/Subacute Form (*n* = 11)	Chronic Form (*n* = 25)	Acute/Subacute Form (*n* = 22)	Chronic Form (*n* = 50)
Fever	7 63.6%	1 * 4%	19 86.4%	18 * 36%
Weight Loss	10 * 90.9%	13 52%	10 * 45.5%	37 74%
Jaundice	5 45.5%	0 0%	4 18.2%	1 2%
Lymph Node Involvement Number of affected chains/patients ^a^	11 100% 3.9 ± 1.6	5 20% 0.3 ± 0.7	20 90.9% 3.3 ± 1.9	19 38% 0.6 ± 1
Hepatomegaly	7 63.6%	2 8%	10 45.5%	4 8%
Splenomegaly	6 54.4%	0 0%	7 31.8%	3 6%
Skin Lesion	6 54.5%	3 * 12%	9 40.9%	22 * 44%
Ulceration of the Oral/Nasal Mucosa	1 9%	10 40%	5 22.7%	26 52%
Larynx Involvement	0 0%	6 24%	1 4.5%	9 18%
Lung Injury	3 27.3%	20 80%	4 18.2%	41 82%
Bone Lesion	0 0%	2 8%	0 0%	2 4%
CNS Injury	0 0%	1 4%	0 0%	3 6%

* *p* < 0.01. ^a^ Mean ± Std. Deviation; chains considered: cervical, supraclavicular, axillary, thoracic, abdominal, and inguinal.

**Table 3:** Laboratory markers associated with the severity of disease in women and men with paracoccidioidomycosis.

	FEMALE		MALE	
Laboratory Tests	Acute/Subacute Form	Chronic Form	Acute/Subacute Form	Chronic Form
Anemia	11/11 100%	5/25 20%	15/22 61.2%	16/48 33.4%
Leukocytosis	4/11 36.4%	5/25 20%	5/22 22.7%	16/48 33.3%
Leukopenia	1/11 9%	0/35 0%	0/22 0%	0/48 0%
Eosinophilia	6/11 54.5%	3/25 ** 12%	10/22 45.5%	19/48 ** 39.6%
Hypoalbuminemia	8/9 88.9%	0/15 ** 0%	11/17 64.7%	11/22 ** 50%
Hypergammaglobulinemia	7/9 77.8%	7/15 46.7%	15/17 88.2%	9/22 40.9%
Gamma:Albumin Ratio	0.83 (0.4–1.6)	0.36 (0.2–0.7)	1 (0.2–2.6)	0.57 (0.2–1)
C-Reactive Protein	8/9 88.9%	11/20 * 55%	14/15 93.3%	29/33 * 87.9%
Erythrocyte Sedimentation Rate	5/6 83.3%	9/10 90%	5/6 83.3%	22/27 81.5%

* *p* <0.01, ** *p* < 0.001.

**Table 4:** Abnormalities of CF women’s and men’s pulmonary HRCTs taken before treatment.

HRCT Features	Female (*n* = 13)	Male (*n* = 21)
Nodules	10 77%	15 71%
Ground-Glass Opacities	12 * 92%	11 * 52%
Micronodules	9 69%	13 62%
Paraseptal/Centrolobular Emphysema	4 * 31%	14 * 67%
Bronchial Wall Thickening	7 54%	11 52%
Parenchymal Bands	4 31%	12 57%
Interlobular Septal Thickening and/or Reticulation	6 46%	8 38%
Mediastinal Lymphadenopathy	4 31%	10 48%
Paracicatricial Emphysema	2 15%	9 43%
Cavities	4 31%	6 29%
Architectural Distortion	1 8%	5 24%
Consolidation	3 23%	3 14%
Pleural Effusion	1 8%	2 10%
Pleural Thickening	0 0%	2 10%

* *p* < 0.05.

## Data Availability

The data presented in this study are available on request from the corresponding author. The data are not publicly available due to ethical restrictions.
